# Deep learning reconstruction accelerated reduced field-of-view DWI in rectal cancer: mucosa-submucosa-muscularis visualization and T staging

**DOI:** 10.1186/s41747-025-00667-x

**Published:** 2026-01-26

**Authors:** Wenjing Peng, Fan Yang, Diliang Li, Rui Zhao, Lijuan Wan, Shuang Chen, Xiangchun Liu, Sicong Wang, Yuanlong Li, Min Li, Yuan Liu, Hongmei Zhang

**Affiliations:** 1 Department of Radiology, National Cancer Center/National Clinical Research Center for Cancer/Cancer Hospital, Chinese Academy of Medical Sciences and Peking Union Medical College, Beijing, China; 2 GE Healthcare, Beijing, China

**Keywords:** Deep learning, Diffusion magnetic resonance imaging, Image processing (computer-assisted), Neoplasm staging, Rectal neoplasms

## Abstract

**Objective:**

We compared the image quality and diagnostic performance of deep learning reconstruction (DLR) accelerated reduced field-of-view (rFOV_DL_) diffusion-weighted imaging (DWI) with standard-reconstructed full field-of-view (fFOV_STA_) DWI in rectal cancer.

**Materials and methods:**

This prospective study enrolled 173 participants with biopsy-confirmed rectal adenocarcinoma from November 2022 to August 2023 undergoing rFOV_DL_ and fFOV_STA_ DWI scans. Two radiologists evaluated qualitative image quality, objective image quality, and apparent diffusion coefficient (ADC) independently. T and N staging were evaluated in 94 participants undergoing radical surgery. Diagnostic sensitivity, specificity, and accuracy were calculated using histopathologic results as the gold standard. ADC values were analyzed for correlations with histopathologic staging.

**Results:**

We observed that rFOV_DL_ DWI reduced acquisition time by 30% compared to fFOV_STA_ DWI. rFOV_DL_ DWI outperformed fFOV_STA_ DWI in all qualitative image quality metrics (*p* ≤ 0.013), especially in mucosa-submucosa-muscularis visualization, spatial resolution, overall image quality, and diagnostic confidence, accompanied by comparable objective image quality (*p* ≥ 0.054). When applied with T2-weighted imaging, rFOV_DL_ DWI significantly enhanced primary T-staging accuracy than fFOV_STA_ DWI (*p* < 0.001), especially for early-stage tumors (T1 or T2). Tumor ADC values of rFOV_DL_ DWI were lower than those of fFOV_STA_ DWI, yet remained solid inverse correlations with histopathologic T-staging (*p* < 0.001). Higher inter-reader agreements of locoregional staging and ADC measurements were obtained by rFOV_DL_ DWI.

**Conclusion:**

rFOV_DL_ DWI significantly improved image quality than fFOV_STA_ DWI, with a 30% reduced acquisition time. rFOV_DL_ DWI facilitated higher primary T-staging accuracy, especially for early-stage rectal cancer (T1–T2).

**Relevance statement:**

Reduced acquisition time and improved imaging quality highlighted the clinical feasibility of applying DLR to rFOV DWI. rFOV_DL_ DWI could significantly enhance primary T-staging accuracy, especially for early-stage rectal cancer (T1–T2), facilitating more precise treatment management.

**Key Points:**

- Applying deep learning reconstruction (DLR) to reduced field-of-view (rFOV) diffusion-weighted imaging (DWI) improved mucosa-submucosa-muscularis visualization and reduced acquisition time.
- DLR-based rFOV DWI significantly enhanced primary T-staging accuracy for rectal cancer, especially for early-stage tumors (T1 or T2).
- DLR-based rFOV DWI facilitated higher inter-reader agreements for locoregional staging and apparent diffusion coefficient measurement in rectal cancer.

**Graphical Abstract:**

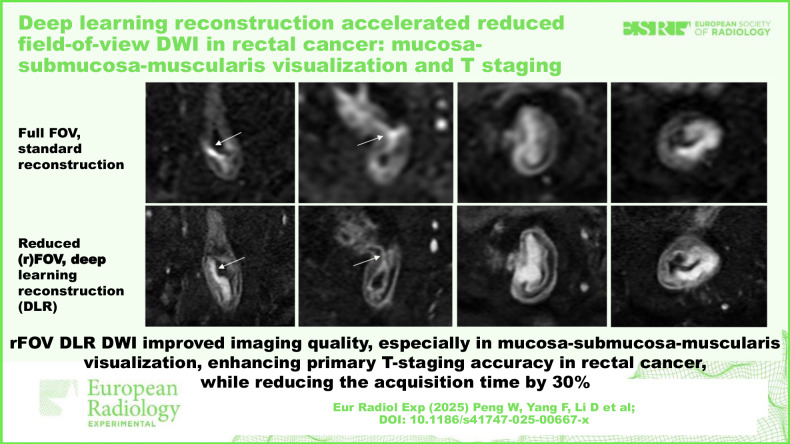

## Background

Colorectal cancer is the third most prevalent cancer globally, with rectal cancer accounting for approximately one-third of cases [1]. Precise pretreatment staging is a critical foundation for selecting treatment strategies and assessing prognosis. Currently, magnetic resonance imaging (MRI) is the primary imaging examination for assessing rectal cancer, with high-resolution T2-weighted imaging serving as the first-line modality [2, 3]. However, great challenges remain in clinical practice. Overstaging rates as high as 69–95% have been reported for T1 tumors assessed by MRI [4–6], which may lead to excessive treatment. Although guidelines recommend endorectal ultrasound as a supplementary examination [2], this approach may increase healthcare costs. Its field of view (FOV) is also limited in the comprehensive assessment of lymph nodes, and its diagnostic efficiency largely depends on the operator’s experience. Moreover, accurate differentiation between extramural desmoplastic reactions and tumor invasion remains a notable issue in rectal cancer, frequently leading to the misclassification of T2 and early T3 tumors [7, 8], which might affect individualized treatment decisions. Therefore, improving the initial staging accuracy is of great significance for rectal cancer.

Diffusion-weighted imaging (DWI) holds the potential for enhancing the performance of T2-weighted imaging, such as better detecting residual tumors after neoadjuvant chemoradiotherapy (nCRT) [9–11], while its value in the primary staging of rectal cancer remains uncertain. This was primarily attributed to the suboptimal image quality of DWI according to the literature [12–14]. Full FOV (fFOV) single-shot echo-planar imaging is the most commonly used DWI technique. But it shows low spatial resolution due to the narrow bandwidth in the phase-encoding direction and is also prone to image distortion, blurring, and susceptibility artifacts [15–17]. Reduced FOV (rFOV) DWI is a modified modality that can enhance spatial resolution by decreasing the effective phase-encoding FOV and encoding steps required to fill k-space. It has been demonstrated to be effective in reducing artifacts and blurring in multiple organ systems [15, 18, 19]. In rectal MRI, rFOV DWI has proven superior image quality compared to traditional DWI, facilitating enhanced accuracy in assessing complete response after nCRT [20]. However, a standard rFOV DWI requires a scanning time of approximately 4–5 min [20, 21], and the smaller FOV often results in a reduced signal-to-noise ratio (SNR). These limitations hinder its widespread adoption in clinical practice.

Deep learning reconstruction (DLR) is an advanced image-enhanced technique. Different from most conventional deep learning–based denoising methods, which work in the image domain on fully reconstructed images, DLR applies convolutional neural networks directly in the raw k-space domain [22]. This allows noise suppression before image reconstruction, preserves fine structural details, and produces higher-quality images from low-quality acquisitions. By far, DLR has been successfully applied to various anatomical MRI scans [23, 24]. Recent studies have also successively verified its effectiveness in accelerated rectal T2-weighted imaging [25, 26], highlighting its potential in improving initial staging efficacy. Nevertheless, little attention has been given to the application of DLR in rFOV DWI. We hypothesize that integrating DLR with rFOV DWI could enhance image quality with shortened scan time and facilitate more precise locoregional staging in rectal cancer.

Therefore, this study aimed to compare the image quality and diagnostic performance between DLR-accelerated rFOV DWI and conventional fFOV DWI for primary locoregional staging of rectal cancer.

## Materials and methods

This was a prospective study and received approval from the institutional review board (No. NCC2021C-456). The entire study was conducted in accordance with the ethical principles outlined in the Declaration of Helsinki. All participants were fully informed and signed the written informed consent form before the MRI examination.

### Study participants

**Fig. 1:**
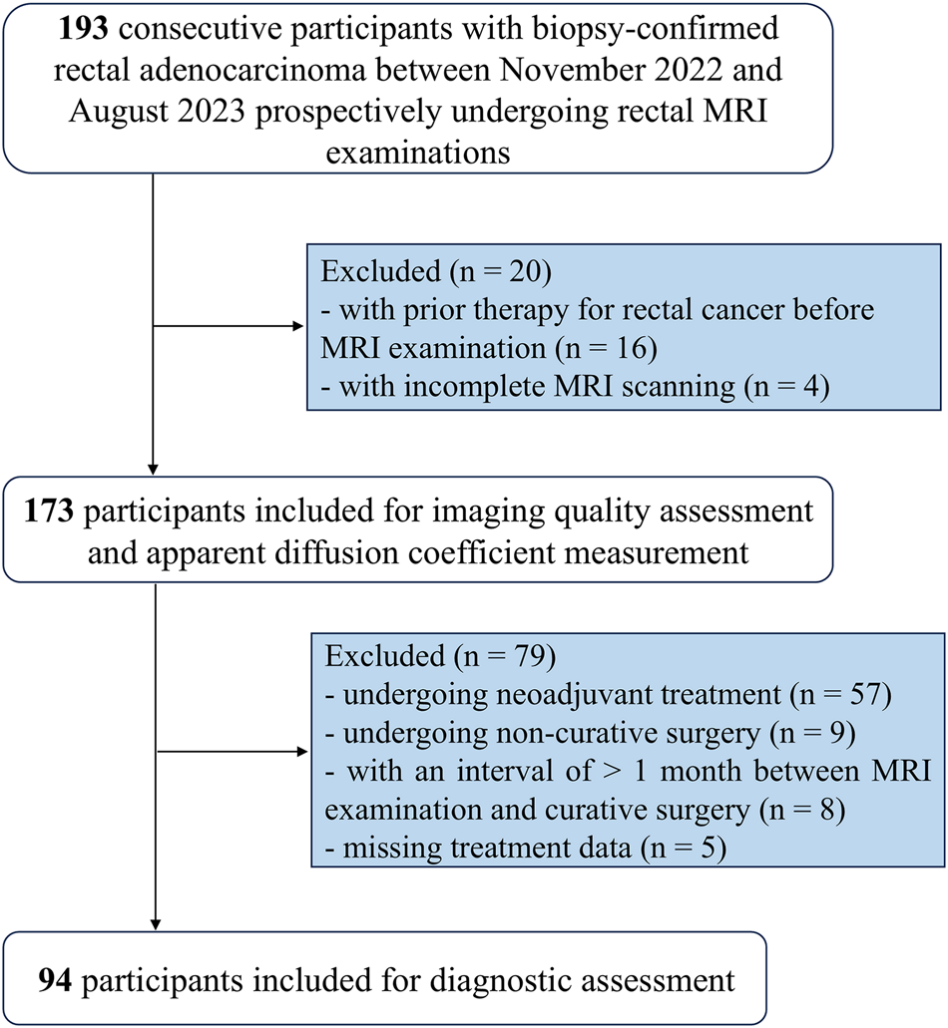
Study flowchart

This study prospectively recruited consecutive participants with biopsy-proven rectal adenocarcinoma at our institution between November 2022 and August 2023 to undergo rectal MRI scans (*n* = 193). All MRI data were enrolled for image quality and apparent diffusion coefficient (ADC) analysis, except those who had received prior treatment for rectal cancer (*n* = 16) or failed to complete MRI scanning (*n* = 4). Re-selected participants receiving primary curative surgery underwent further locoregional staging assessment. Participants undergoing neoadjuvant therapy (*n* = 57), undergoing non-radical surgery (*n* = 9), with over 1-month interval between surgery and MRI scan (*n* = 8), and with missing treatment data (*n* = 5) were excluded. Ultimately, 173 participants were included for imaging quality assessment and ADC measurement, and 94 for primary locoregional staging assessment. The largest lesion was assessed for participants with multifocal tumors. The study flowchart is illustrated in Fig. 1.

### MRI acquisition

All scans were conducted employing a 3.0-T MRI scanner (SIGNA Architect; GE Healthcare), utilizing a thirty-channel AIR™ receiver array, positioned supine. Participants adopted a glycerin enema and 10 mg intramuscular injection of raceanisodamine hydrochloride (unless contraindicated) before scanning. Sagittal T2-weighted imaging, oblique axial and coronal small FOV T2-weighted imaging perpendicular and parallel to tumor axis; axial non-fat-saturated T1-weighted imaging, axial fat-saturated T2-weighted imaging, and axial, sagittal, and coronal contrast-enhanced T1-weighted imaging, were performed according to the European Society of Gastrointestinal and Abdominal Radiology [3]. Two sets of oblique axial DWI perpendicular to the tumor axis were designed for comparison, including rFOV DWI reconstructed by deep learning protocol (rFOV_DL_ DWI) and single-shot echo-planar fFOV DWI reconstructed by standard protocol (fFOV_STA_ DWI). rFOV DWI was facilitated by a spatially selective excitation scheme, integrating a 2D echo-planar radiofrequency excitation pulse with a 180° refocusing pulse. Detailed DWI parameters were as follows: fFOV_STA_ DWI had a repetition time of 4950 ms, echo time of 73 ms, field of view of 400 × 320 mm, matrix size of 140 × 160, number of excitations of 8, a bandwidth of 250 Hz/pixel; rFOV_DL_ DWI had a repetition time of 6350 ms, echo time of 73 ms, field of view of 180 × 90 mm, matrix size of 128 × 128, number of excitations of 4, a bandwidth of 250 Hz/pixel. They shared the same slice thickness of 5 mm, intersection gap of 1 mm, total slice of 24, and b-values of 0 and 1000 s/mm². The acquisition time for fFOV_STA_ DWI was 2 min 18 s, whereas rFOV_DL_ DWI required 1 min 36 s. Specifically, this study achieved acceleration by reducing the number of excitations in rFOV DWI to shorten the acquisition time. DLR was subsequently applied for k-space–based image enhancement to compensate for the resulting SNR loss, including denoising and de-aliasing. The non-DLR rFOV DWI sequence was not prospectively acquired in this study due to the consideration of prolonged reconstruction time, additional burden on data transfer and storage, and potential challenges to routine clinical workflow. Our primary aim was focused on evaluating the clinical applicability of the rFOV_DL_ DWI, as compared with conventional fFOV_STA_ DWI.

A vendor-supplied DLR algorithm (AIR™ Recon DL, GE Healthcare, Waukesha, WI, USA) was seamlessly integrated into the scanner’s proprietary image processing framework, enabling online reconstruction. The DLR model was trained in a supervised manner on a database of over 4.4 million pairs of artifact-free, high-SNR, high-spatial-resolution images and their corresponding low-SNR, low-spatial-resolution images. The training data were highly diverse, including various anatomies, scanning parameters, field strengths (1.5 or 3 T), and a broad range of subject characteristics. In addition, multiple data augmentation strategies were applied to enhance the model’s robustness and minimize the risk of overfitting. Although there is no publicly available information indicating that the model has been retrained or validated specifically for rectal DWI, it has undergone independent validation across multiple MRI sequences, phantoms, and *in vivo* imaging of various anatomical sites, demonstrating its broad applicability. A more detailed description of the model construction and validation process is provided in the white paper [22]. During post-processing, three configurable denoising intensities can be selected: low (25% noise reduction), medium (50% noise reduction), and high (75% noise reduction). Multiple phantom studies have demonstrated that high-level denoising yields the highest SNR and image sharpness while preserving structural details [27, 28]. Previous clinical studies across various organs have also confirmed the robustness and reliability of high-level denoising in real-world practice [21, 25, 29]. Given that the raw rFOV DWI images analyzed in this study exhibited a high noise level and that low or moderate denoising intensities might be insufficient to compensate for the SNR loss, we adopted a high-level denoising intensity.

### Image quality assessment and ADC measurement

Two radiologists (Y.L. and W.J.P., a senior and a junior radiologist specializing in rectal MRI for 20 and 5 years, respectively) performed the image quality and ADC assessment independently on the workstation. The image assessments of the two DWI sequences were conducted separately, with all sequence names and reconstruction types anonymized to minimize subjective bias. Following experience from previous studies [30–32] and taking into account the nature of DWI reading tasks in this study, a minimum washout period of 3 weeks was implemented between two sessions, with the reading order randomized to minimize recall bias. Throughout the evaluation, both radiologists were blinded to any clinical and pathological information of the patients.

The qualitative assessment of image quality primarily focused on the anatomical visualization of the bowel wall and tumor lesions, including mucosa-submucosa–muscularis visualization, lesion conspicuity, edge sharpness, and spatial resolution. In addition, artifacts, distortion, overall image quality, and diagnostic confidence were also evaluated. All of the qualitative parameters were scored based on a 5-point Likert scale: 1, inadequate for diagnosis; 2, poor quality for diagnosis; 3, acceptable for moderate diagnosis; 4, good for adequate diagnosis; and 5, excellent for definitive diagnosis. In addition, three objective image quality parameters, including SNR of the internal obturator muscle (SNR_internal obturator muscle_), SNR of the tumor (SNR_tumor_), and contrast-to-noise ratio (CNR) between tumor and internal obturator muscle, were measured synchronously [24]. Detailed extraction methods are provided in Supplement S1 (online). ADC maps of two DWI sequences were generated using mono-exponential fitting, and tumor ADC values were measured using the same method. Prior to the formal evaluation, we conducted a dedicated, systematic reader training to maximize the objectivity and rigor of blinded image assessment. The training was based on images from 20 independent patients with rectal cancer and emphasized consistent and objective scoring criteria as well as standardized interpretation procedures. Readers were repeatedly reminded to focus on image quality and diagnostic features, while avoiding recognition of the sequence type or reconstruction method, in order to minimize subjective bias arising from differences in image appearance. They then independently reviewed the images following the same protocol and subsequently resolved discrepancies through joint review to refine the evaluation standards.

Inter-reader agreements were assessed across the entire cohort by comparing the results from two readers. Intra-reader agreement was evaluated through repeated assessments in 50 randomly selected participants. A washout period of at least 3 weeks was also maintained between two reading sessions to minimize recall bias.

### Primary locoregional staging assessment

Participants who underwent primary radical surgery were involved in primary staging evaluation, including T-staging (Tis–T4) and N-staging (positive or negative), using histopathologic results as the reference standards. The same two readers as the imaging quality analysis conducted this diagnostic work independently. High-resolution oblique axial T2-weighted imaging and two separate DWI sequences served as the primary diagnostic series, with standard-reconstructed sagittal and coronal T2-weighted imaging as the supplementary reference. Prior to the formal assessments, both readers received centralized training in rectal MRI interpretation to ensure standardization. Following a similar procedure to the image quality analysis, diagnostic evaluations of two DWI were conducted separately. The order of cases was randomized for each session, and a minimum 3-week washout period was applied to reduce recall bias. Readers were blinded to the DWI reconstruction type and any clinical or pathological information. To allow for a clearer conclusion of the influence of different DWI sequences on the initial staging of rectal cancer, a co-reading from two radiologists was also conducted after an independent session. Specifically, co-reading refers to a consensus reading approach. Two radiologists independently assessed the images according to a standardized scoring system. For cases with discrepancies, they conducted a joint review, thoroughly discussing the anatomical and lesion characteristics, and ultimately reached a consensus for staging.

Inter-reader agreement in primary staging was evaluated based on the independent assessments from two readers.

### Histopathologic results

Total mesorectal excision was performed as the standard surgical approach. Histopathologic evaluation of the resected specimens was conducted by two expert pathologists, blinded to the radiological findings. The 8th American Joint Committee on Cancer TNM classification system [33] served as the reference standard.

### Statistical analysis

All data were analyzed using IBM SPSS Statistics (version 25.0), with statistical significance set at *p* < 0.05. Descriptive statistics were used to summarize the clinical or demographic data. The Kolmogorov–Smirnov test was employed to assess the normality for continuous variables. Imaging quality parameters and ADC values of rFOV_DL_ and fFOV_STA_ DWI were compared using the paired-sample *t*-test or Wilcoxon signed-rank test. Diagnostic performances, including the overall T-staging accuracy, sensitivity, specificity, and accuracy of the T sub-stage and N stage were compared using McNemar’s test. Spearman’s rank and Point-biserial correlation analysis were employed to examine the relationship between ADC values and histopathologic staging. Intra- and inter-reader agreements for qualitative image quality scoring and inter-reader agreements for diagnostic performance were evaluated by Cohen κ. Intra- and inter-reader agreements for objective imaging quality metrics were evaluated by the intraclass correlation coefficient (ICC). Their interpretations were as follows: for κ statistics, 0–0.20 (poor), 0.21–0.40 (fair), 0.41–0.60 (moderate), 0.61–0.80 (good), and 0.81–1.00 (excellent); for ICC, 0–0.49 (poor), 0.50–0.75 (moderate), 0.76–0.90 (good), and 0.91–1.00 (excellent) [34].

## Results

### Study participants

A total of 173 participants (mean age, 60 ± 11 years; 109 men) with rectal adenocarcinoma were enrolled for imaging quality assessment and ADC measurements, among whom 94 (mean age, 59 ± 11 years; 55 men) underwent primary radical surgery for further diagnostic performance (Fig. 1). The participant demographic and clinical characteristics are summarized in Table 1. Based on surgical histopathology, tumors were classified as follows: Tis/T1 (*n* = 10, 11%), T2 (*n* = 26, 28%), T3 (*n* = 46, 49%), and T4 (*n* = 12, 13%). Lymph node metastasis (N+) was present in 45 patients (48%).

**Table 1:** Demographic and clinical characteristics of participants

Characteristic	All participants *(n* = 173)	Surgery participants (*n* = 94)
Age (years)*	60 ± 11	59 ± 11
Sex		
Male	109 (63)	55 (59)
Female	64 (37)	39 (41)
Pretreatment CEA level		
≤ 5 ng/mL	98 (57)	64 (68)
> 5 ng/mL	67 (39)	29 (31)
Missing	8 (5)	1 (1)
Pretreatment CA19-9 level		
≤ 30 U/mL	136 (79)	81 (86)
> 30 U/mL	28 (16)	11 (12)
Missing	9 (5)	2 (2)
Tumor location		
Upper	27 (16)	22 (23)
Medium	59 (34)	31 (33)
Low	87 (50)	41 (44)
Participants with multifocal lesions	6 (3)	3 (3)
Surgical procedures		
Dixon	—	79 (84)
Miles	—	12 (13)
Hartman	—	3 (3)

Unless otherwise indicated, data are numbers of patients, with percentages in parentheses

*CA19-9* Carbohydrate antigen 19-9, *CEA* Carcinoembryonic antigen

* Data are mean ± standard deviation

DLR reduced rFOV DWI’s scan time by 30% compared to fFOV_STA_ DWI.

### Image quality assessment

**Fig. 2:**
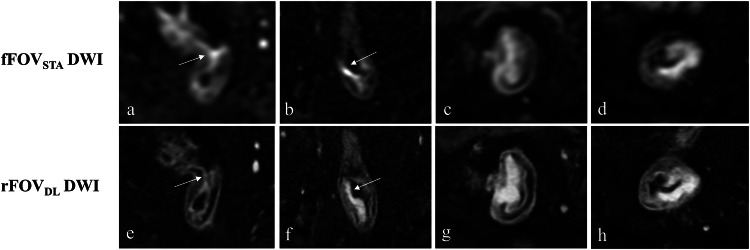
Representative cases displaying the enhanced qualitative imaging quality of rFOV_DL_ DWI compared to fFOV_STA_ DWI in four patients with rectal cancer, manifested by reduced susceptibility artifacts (**e** *versus* **a**, arrows), reduced distortion (**f** *versus* **b**, arrows), improved lesion conspicuity (**f**–**h** *versus* **b**–**d**), and improved edge sharpness (**f**–**h** *versus* **b**–**d**). All the rFOV_DL_ DWI (**e**–**h**) exhibited enhanced spatial resolution, mucosa-submucosa-muscularis visualization, overall image quality, and diagnostic confidence than fFOV_STA_ DWI (**a**–**d**). DWI, Diffusion-weighted imaging; fFOV_STA_, Standard-reconstructed full field-of-view; rFOV_DL_, Deep learning-reconstructed reduced field-of-view

**Fig. 3:**
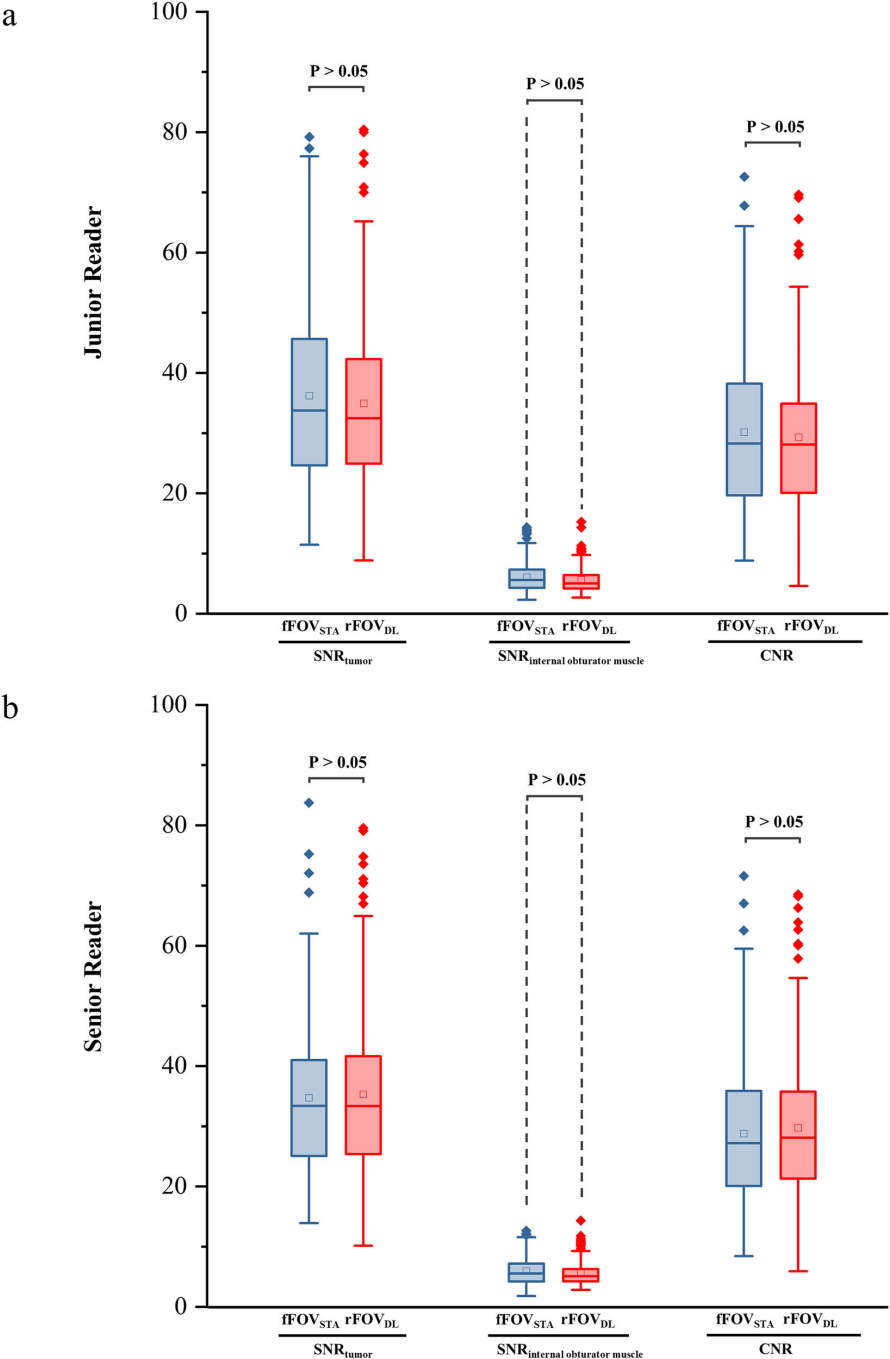
Box plots showing the comparison of objective image quality metrics between fFOV_STA_ and rFOV_DL_ DWI, assessed by junior (**a**) and senior (**b**) readers in the entire cohort (*n* = 173). 工 indicates 1.5*IQR, 一 indicates medians, □ indicates means, ◆ indicates abnormal values. CNR, Contrast-to-noise ratio; DWI, Diffusion-weighted imaging; fFOV_STA_, Standard-reconstructed full field-of-view; rFOV_DL_, Deep learning-reconstructed reduced field-of-view; SNR, Signal-to-noise ratio

Descriptive statistics of the qualitative image quality scoring are presented in Table 2. Both the junior and senior radiologists reached consistent conclusions that rFOV_DL_ DWI exhibited significantly improved mucosa-submucosa-muscularis visualization, spatial resolution, edge sharpness, and lesion conspicuity compared to fFOV_STA_ DWI, accompanied by reduced artifacts and distortion (all *p* < 0.05). rFOV_DL_ DWI outperformed fFOV_STA_ DWI in overall image quality and diagnostic confidence (all *p* < 0.05). Figure 2 illustrates representative cases of qualitative imaging quality assessment. No significant differences were observed in the objective imaging quality between rFOV_DL_ DWI and fFOV_STA_ DWI: SNR_tumor_ (junior reader, 34.95 ± 13.49 *versus* 36.21 ± 14.78, *p* = 0.439; senior reader, 35.33 ± 13.62 *versus* 34.72 ± 13.01, *p* = 0.700), SNR_internal obturator muscle_ (junior reader, 5.63 ± 2.09 *versus* 6.05 ± 2.43, *p* = 0.054; senior reader, 5.59 ± 1.94 *versus* 5.94 ± 2.22, *p* = 0.058), and CNR (junior reader, 29.32 ± 12.07 *versus* 30.16 ± 13.15, *p* = 0.520; senior reader, 29.73 ± 12.45 *versus* 28.79 ± 11.99, *p* = 0.436). Figure 3 displays the comparison of objective imaging quality between rFOV_DL_ DWI and fFOV_STA_ DWI.

**Table 2:** Comparison of qualitative image quality metrics between fFOV_STA_ and rFOV_DL_ DWI

	fFOV_STA_ DWI	rFOV_DL_ DWI	*p*-value
Junior reader			
Artifacts	3.85 ± 0.69	4.05 ± 0.62	0.009
Distortion	3.78 ± 0.62	4.17 ± 0.69	< 0.001
Lesion conspicuity	4.54 ± 0.71	4.76 ± 0.51	0.013
Edge sharpness	3.99 ± 0.70	4.74 ± 0.48	< 0.001
Spatial resolution	3.32 ± 0.47	4.58 ± 0.53	< 0.001
Mucosa-submucosa-muscularis visualization	3.36 ± 0.52	4.55 ± 0.58	< 0.001
Overall image quality	3.48 ± 0.57	4.56 ± 0.64	< 0.001
Diagnostic confidence	3.51 ± 0.54	4.58 ± 0.61	< 0.001
Senior reader			
Artifacts	3.85 ± 0.62	4.05 ± 0.54	0.007
Distortion	3.79 ± 0.59	4.16 ± 0.64	< 0.001
Lesion conspicuity	4.54 ± 0.71	4.79 ± 0.49	0.004
Edge sharpness	3.95 ± 0.66	4.73 ± 0.48	< 0.001
Spatial resolution	3.33 ± 0.47	4.60 ± 0.50	< 0.001
Mucosa-submucosa-muscularis visualization	3.37 ± 0.52	4.56 ± 0.57	< 0.001
Overall image quality	3.51 ± 0.56	4.60 ± 0.63	< 0.001
Diagnostic confidence	3.53 ± 0.52	4.62 ± 0.61	< 0.001

Descriptive data are mean ± standard deviation

*DWI* Diffusion-weighted imaging, *fFOV*_*STA*_ Standard-reconstructed full field-of-view, *rFOV*_*DL*_ Deep learning-reconstructed reduced field-of-view

rFOV_DL_ DWI achieved comparable or higher intra-reader agreement (κ = 0.89–1) in subjective image quality assessments compared to fFOV_STA_ DWI (κ = 0.84–0.93). For inter-reader agreement, rFOV_DL_ DWI demonstrated comparable or higher consistency than fFOV_STA_ DWI in terms of mucosa-submucosa–muscularis visualization, spatial resolution, edge sharpness, overall image quality, and diagnostic confidence (κ = 0.84–0.90 *versus* 0.77–0.85). No notable advantage was observed in artifacts, distortion, and lesion conspicuity (κ = 0.79–0.88 *versus* 0.83–0.90). rFOV_DL_ DWI also demonstrated higher intra-reader (ICC = 0.85–0.92 *versus* 0.81–0.87) and inter-reader agreement (ICC = 0.74–0.83 *versus* 0.73–0.78) in objective quality assessments compared to fFOV_STA_ DWI (Table 3).

**Table 3:** Intra- and inter-reader agreements for image quality assessment and ADC measurement

Characteristic	Intra-reader agreement				Inter-reader agreement	
	Junior reader		Senior reader			
	fFOV_STA_ DWI	rFOV_DL_ DWI	fFOV_STA_ DWI	rFOV_DL_ DWI	fFOV_STA_ DWI	rFOV_DL_ DWI
Qualitative image quality parameters						
Artifacts	0.89	0.89	0.88	0.91	0.84	0.83
Distortion	0.93	0.94	0.92	0.93	0.90	0.88
Lesion conspicuity	0.87	0.94	0.90	0.94	0.83	0.79
Edge sharpness	0.86	0.90	0.84	0.95	0.77	0.86
Spatial resolution	0.91	0.96	0.92	1	0.80	0.90
Mucosa-submucosa-muscularis visualization	0.93	0.96	0.92	0.96	0.81	0.84
Overall image quality	0.90	0.93	0.89	0.97	0.85	0.85
Diagnostic confidence	0.85	0.93	0.84	0.92	0.83	0.87
Objective image quality parameters						
SNR_tumor_	0.86	0.92	0.87	0.91	0.78	0.83
SNR_internal obturator muscle_	0.84	0.85	0.81	0.86	0.73	0.74
CNR	0.85	0.90	0.86	0.90	0.77	0.83

The intra- and inter-reader agreements were assessed using the intraclass correlation coefficient or Cohen κ. The intra-reader agreements were obtained based on the re-assessment of 50 participants randomly selected from the cohort

*ADC* Apparent diffusion coefficient, *CNR* Contrast-to-noise ratio, *DWI* Diffusion-weighted imaging, *fFOV*_*STA*_ Standard-reconstructed full field-of-view, *rFOV*_*DL*_ Deep learning-reconstructed reduced field-of-view, *SNR* Signal-to-noise ratio

### ADC value and correlations with histopathologic staging

Tumor ADC values derived from rFOV_DL_ DWI were smaller than those of fFOV_STA_ DWI (junior reader, 0.908 ± 0.143 *versus* 0.962 ± 0.160 × 10^-3^ mm^2^/s; senior reader, 0.912 ± 0.144 *versus* 0.967 ± 0.151 × 10^-3^ mm^2^/s, both *p* < 0.001, Table S1). However, they exhibited solid inverse correlations with histopathologic T staging (junior reader, *r* = -0.433; senior reader, *r* = -0.379, both *p* < 0.001), which was comparable to those of fFOV_STA_ DWI (junior reader, *r* = -0.446, *p* < 0.001; senior reader, *r* = -0.330, *p* = 0.001). No significant correlations were observed between ADC values and N staging either from fFOV_STA_ or rFOV_DL_ DWI (all *p* > 0.05).

rFOV_DL_ DWI exhibited higher intra-reader agreements (junior reader, κ = 0.89 *versus* 0.82; senior reader, κ = 0.88 *versus* 0.83) and inter-reader agreements (κ = 0.85 *versus* 0.77) than fFOV_STA_ DWI.

### Primary locoregional staging assessment

**Fig. 4:**
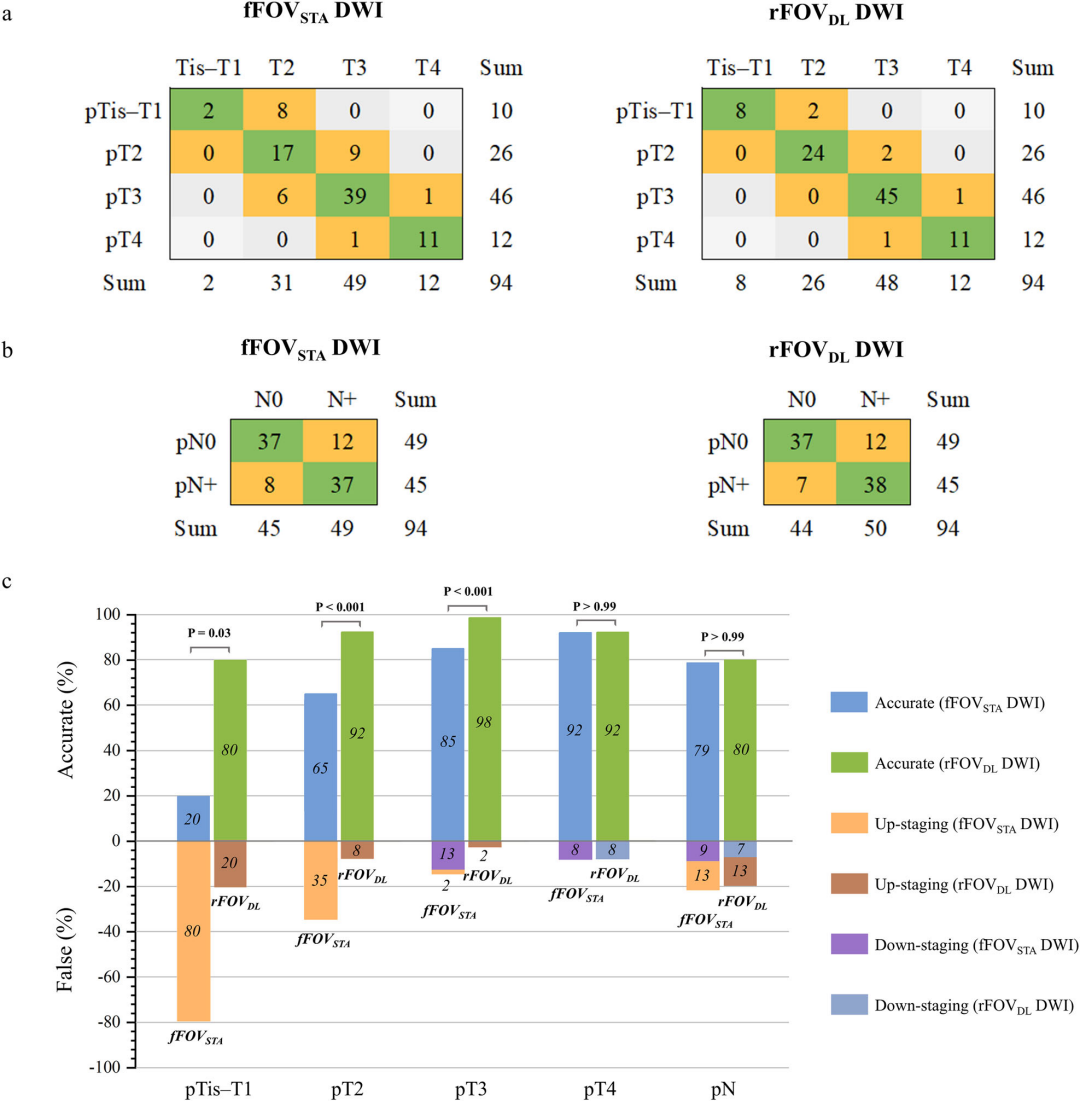
Locoregional staging performance of rFOV_DL_ and fFOV_STA_ DWI, when applied with T2-weighted imaging, according to co-reading results. Confusion matrices show the comparison between clinical T (**a**) and N stage (**b**) with histopathologic results. Bar plots (**c**) show the accurate (above the *x*-axis) and false rates (up-staging and down-staging, below the *x*-axis) for each T and N staging. DWI, Diffusion-weighted imaging; fFOV_STA_, Standard-reconstructed full field-of-view; rFOV_DL_, Deep learning-reconstructed reduced field-of-view

**Fig. 5:**
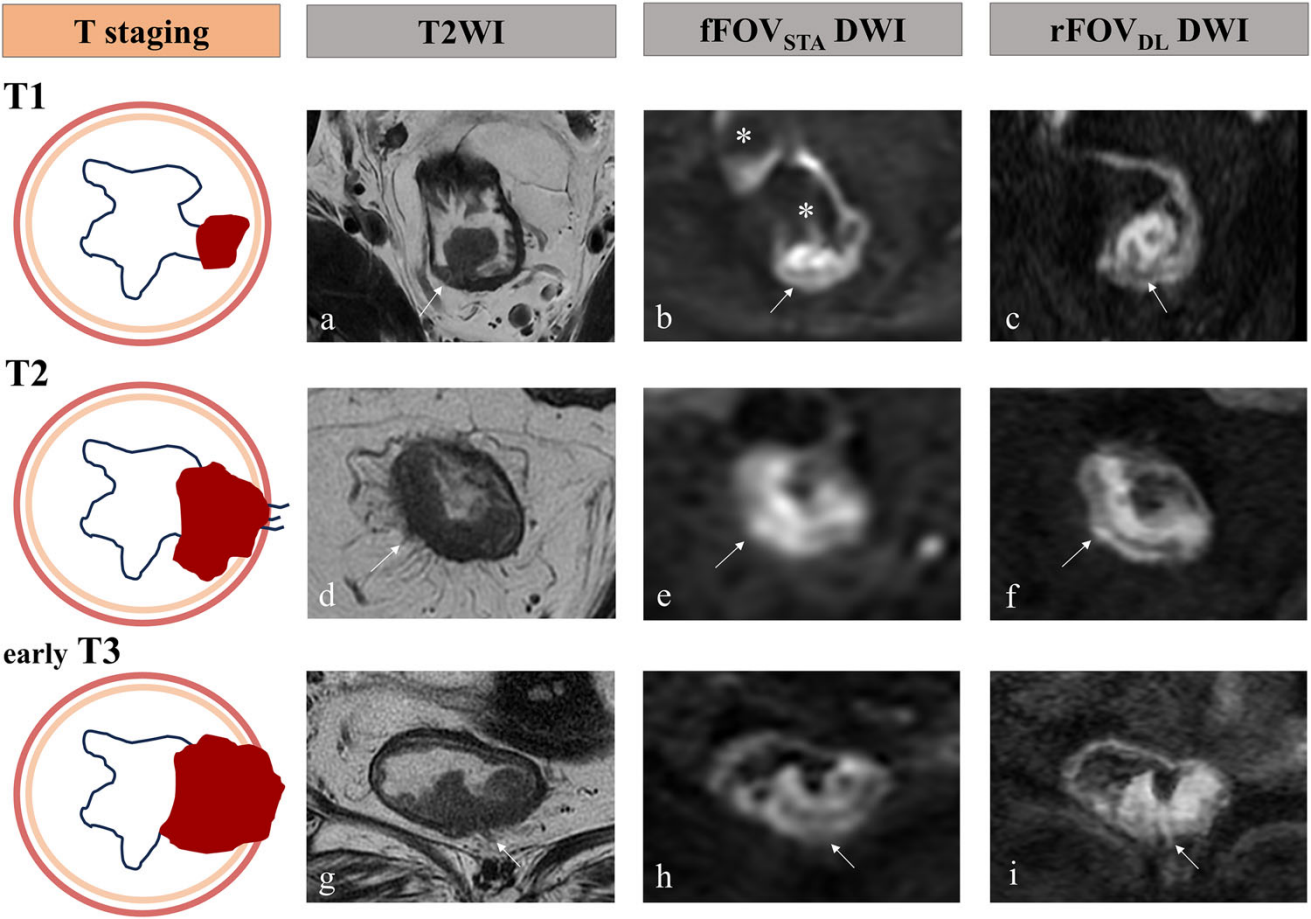
Examples showing the improved primary T-staging accuracy of rFOV_DL_ DWI compared to fFOV_STA_ DWI in three patients with rectal cancer. For patient 1 (**a**–**c**): T2WI (**a**) revealed a pedunculated adenomatous lesion (arrow). fFOV_STA_ DWI (**b**) provided an inconclusive diagnosis due to severe gas-related artifacts (asterisks) and distortion. In contrast, rFOV_DL_ DWI (**c**) exhibited markedly reduced distortion with improved visualization of the rectal wall. It clearly delineated cancerous regions within the adenoma and revealed a preserved low-signal submucosal layer (arrow) between the distal malignant portion and muscularis propria, suggesting T1-stage disease. This staging was subsequently confirmed by histopathology. For patient 2 (**d**–**f**): T2WI (**d**) revealed a nodular protrusion at the muscularis propria periphery (arrow), raising suspicion for extramural invasion. On fFOV_STA_ DWI (**e**), obscured bowel wall layers and a poorly defined high-signal focus (arrow) suggested possible extramural involvement. In contrast, rFOV_DL_ DWI (**f**) provided clearer delineation of tumor margins, revealing the preserved submucosal (arrow) between the tumor and muscularis propria. The remaining part of the tumor locally invaded the muscularis propria, but the whole tumor was limited to the rectal wall, which was consistent with T2-stage disease. Surgical pathology finally confirmed the T2 stage. For patient 3 (**g**–**i**): T2WI (**g**) showed an intramural tumor with smooth outer margins. While a small extramural vessel exhibiting intraluminal enhanced intensity was observed (arrow), the absence of characteristic luminal expansion or irregular contours rendered the diagnosis of EMVI indeterminate. fFOV_STA_ DWI (**h**) demonstrated limited tumor delineation, showing tumor confinement within the bowel wall without conclusive EMVI features (arrow). rFOV_DL_ DWI (**i**) provided superior lesion conspicuity and margin definition, unequivocally demonstrating the irregular outer edge of muscularis propria and definitive EMVI characterized by prominent high-signal-intensity small vessels penetrating beyond the muscular layer (arrow). These findings established a T3-stage and EMVI (+) diagnosis, which were verified by histopathologic results. EMVI, Extramural venous invasion; DWI, Diffusion-weighted imaging; fFOV_STA_, Standard-reconstructed full field-of-view; rFOV_DL_, Deep learning-reconstructed reduced field-of-view; T2WI, T2-weighted imaging

Descriptive statistics illustrating the locoregional staging performance of rFOV_DL_ and fFOV_STA_ DWI are shown in Table 4 and Fig. 4. Both the junior and senior readers consistently found that when combined with T2-weighted imaging, rFOV_DL_ DWI outperformed fFOV_STA_ DWI in overall T-staging accuracy for rectal cancer (fFOV_STA_ *versus* rFOV_DL_: junior reader, 64% *versus* 87%; senior reader, 72% *versus* 94%; co-reading, 73% *versus* 94%, all *p* < 0.001). The most notable improvements were observed in early-stage (Tis–T3) tumors. According to the co-reading results: rFOV_DL_ DWI improved the diagnostic sensitivity of Tis–T1 tumors from 20% with fFOV_STA_ DWI to 80%, with accuracy rising from 91 to 98%. The sensitivity for diagnosing T2 tumors increased from 65 to 92%, with specificity rising from 79 to 97% and accuracy rising from 76 to 96%. For T3 tumors, the sensitivity increased from 85 to 98%, with specificity increasing from 79 to 94% and accuracy increasing from 82 to 96%. Schematic diagram and representative diagnostic cases are illustrated in Fig. 5.

**Table 4:** Comparison of locoregional staging between rFOV_DL_ and fFOV_STA_ DWI

	Junior reader			Senior reader			Co-reading		
	fFOV_STA_ DWI	rFOV_DL_ DWI	*p*-value	fFOV_STA_ DWI	rFOV_DL_ DWI	*p*-value	fFOV_STA_ DWI	rFOV_DL_ DWI	*p*-value
T staging									
Accuracy	64 (60/94)	87 (82/94)	< 0.001*	72 (68/94)	94 (88/94)	< 0.001*	73 (69/94)	94 (88/94)	< 0.001*
Tis–T1									
Sensitivity	10 (1/10)	70 (7/10)	0.031*	20 (2/10)	80 (8/10)	0.031*	20 (2/10)	80 (8/10)	0.031*
Specificity	100 (84/84)	100 (84/84)	**> 0.999**	100 (84/84)	100 (84/84)	**> 0.999**	100 (84/84)	100 (84/84)	**> 0.999**
Accuracy	90 (85/94)	97 (91/94)	0.031*	91 (86/94)	98 (92/94)	0.031*	91 (86/94)	98 (92/94)	0.031*
T2									
Sensitivity	54 (14/26)	88 (23/26)	0.012*	62 (16/26)	92 (24/26)	0.008*	65 (17/26)	92 (24/26)	0.016*
Specificity	76 (52/68)	93 (63/68)	0.001*	79 (54/68)	97 (66/68)	< 0.001*	79 (54/68)	97 (66/68)	< 0.001*
Accuracy	70 (66/94)	91 (86/94)	< 0.001*	74 (70/94)	96 (90/94)	< 0.001*	76 (71/94)	96 (90/94)	< 0.001*
T3									
Sensitivity	80 (37/46)	96 (44/46)	0.016*	85 (39/46)	98 (45/46)	0.031*	85 (39/46)	98 (45/46)	0.031*
Specificity	71 (34/48)	90 (43/48)	0.012*	79 (38/48)	94 (45/48)	0.016*	79 (38/48)	94 (45/48)	0.016*
Accuracy	76 (71/94)	93 (87/94)	< 0.001*	82 (77/94)	96 (90/94)	< 0.001*	82 (77/94)	96 (90/94)	< 0.001*
T4									
Sensitivity	67 (8/12)	67 (8/12)	**> 0.999**	92 (11/12)	92 (11/12)	**> 0.999**	92 (11/12)	92 (11/12)	**> 0.999**
Specificity	95 (78/82)	98 (80/82)	**0.500**	99 (81/82)	99 (81/82)	**> 0.999**	99 (81/82)	99 (81/82)	**> 0.999**
Accuracy	91 (86/94)	94 (88/94)	**0.500**	98 (92/94)	98 (92/94)	**> 0.999**	98 (92/94)	98 (92/94)	**> 0.999**
N staging									
Sensitivity	69 (31/45)	73 (33/45)	**0.625**	76 (34/45)	80 (36/45)	**0.500**	82 (37/45)	84 (38/45)	**> 0.999**
Specificity	69 (34/49)	69 (34/49)	**> 0.999**	73 (36/49)	69 (34/49)	**0.500**	76 (37/49)	76 (37/49)	**> 0.999**
Accuracy	69 (65/94)	71 (67/94)	**0.625**	74 (70/94)	74 (70/94)	**> 0.999**	79 (74/94)	80 (75/94)	**> 0.999**

All the staging results were analyzed based on the surgery participants (*n* = 94), with histopathologic results as the gold standard. The *p*-values for the efficacy comparison are displayed in bold

*DWI* Diffusion-weighted imaging, *fFOV*_*STA*_ Standard-reconstructed full field-of-view, *rFOV*_*DL*_ Deep learning-reconstructed reduced field-of-view

* Data with statistical significance

No statistical differences were observed in the diagnostic performance of T4 stage and N-staging between rFOV_DL_ and fFOV_STA_ DWI (all *p* > 0.05).

rFOV_DL_ DWI demonstrated higher inter-reader agreements for T-staging (κ, 0.91 *versus* 0.76) and N-staging (κ, 0.77 *versus* 0.68) compared to fFOV_STA_ DWI.

## Discussion

This study prospectively verified the clinical feasibility of applying DLR to rFOV DWI. When combined with T2-weighted imaging, rFOV_DL_ DWI significantly improved the initial staging accuracy for rectal cancer, especially for early-stage tumors. DLR enabled a 30% reduction in scan time for rFOV DWI scans, while markedly improving image quality compared to conventional DWI. In addition, rFOV_DL_ DWI facilitated higher inter-reader agreements for locoregional TN staging and ADC measurements.

Compared to conventional DWI, our results demonstrated that DLR-based accelerated rFOV_DL_ DWI achieved superior performance in qualitative image quality metrics, with significantly improved mucosa-submucosa-muscularis visualization, spatial resolution, edge sharpness, and lesion conspicuity, along with reduced artifacts and distortion. Moreover, rFOV_DL_ DWI outperformed fFOV_STA_ DWI in overall image quality and diagnostic confidence. These findings are consistent with prior studies validating the successful application of DLR to accelerated MRI scans [21, 25]. In the context of rectal cancer, enhanced mucosa-submucosa-muscularis visualization is of particular clinical value. Theoretically, DLR excels at enhancing high-low signal contrast by processing complex-valued data with zero-mean noise [35]. On DWI, the rectal wall exhibits a distinct high-low-high signal pattern corresponding to the cellular density in mucosa-submucosa-muscularis layers, which aligns well with DLR’s ability to amplify contrast. Additionally, DLR contributed to improved edge sharpness, further enhancing the rectal wall delineation. Regarding objective image quality parameters, rFOV_DL_ DWI showed no significant differences from fFOV_STA_ DWI, which suggested that DLR effectively compensated for the reduced SNR caused by narrow FOV and shortened acquisition time.

The image quality enhancement achieved with rFOV_DL_ DWI promoted improvements in diagnostic efficacy, with the most relevant clinical benefits seen in guiding management of patients with early-stage tumors (T1 or T2) who are being considered for less invasive treatments. rFOV_DL_ DWI served as an efficient functional sequence to complement T2-weighted imaging in delineating mucosa-submucosa-muscularis stratification. In this context, tumors are less likely to be overstaged, thereby enabling safe consideration of local excision. rFOV_DL_ DWI was even capable of delineating cancerous regions within adenomas, which could guide submucosal excision more safely. Moreover, rFOV_DL_ DWI demonstrated water diffusion restriction more precisely, aiding in the differentiation between desmoplastic reactions and tumor infiltration. This facilitated more accurate identification of T2 tumors and their distinction from early T3 disease. Currently, the role of individualized treatment for T2 *versus* early T3 (T3a/b) rectal cancer remains debated. While several studies suggested that T3a/b tumors may achieve outcomes comparable to T2 with surgery alone, avoiding nCRT [36], certain guidelines, such as the National Comprehensive Cancer Network, remain conservative [2], recommending nCRT for all T3 cases. Furthermore, as modern strategies increasingly emphasize quality of life and organ preservation, precise staging might be increasingly critical. Within this context, our sequence may serve as a robust tool to refine T2/T3 differentiation and facilitate more tailored treatment strategies.

It is noteworthy that the advantages of rFOV_DL_ DWI were not generalized to T4 or N staging. No significant differences were observed in the diagnostic performance of T4 and N staging between fFOV_STA_ and rFOV_DL_ DWI. This aligns with expectations, as precise T4 diagnosis relies more on identifying the anatomical peritoneal reflection; N-staging depends more on detailed descriptions of internal heterogeneity and margin irregularity of the lymph nodes. Despite its improved spatial resolution, rFOV_DL_ DWI had certain limitations in these regards. Nevertheless, the application of rFOV_DL_ DWI improved inter-reader agreements for locoregional T and N staging, which is important for diagnostic reproducibility. Taken together, these findings suggested that rFOV_DL_ DWI held promise of serving as a clinically-feasible, high-resolution DWI modality for clinical practice, offering tangible benefits for the initial staging in rectal cancer.

In this study, the application of DLR not only yielded marked improvements in image quality and diagnostic performance but also enabled a substantial reduction in acquisition time. For patients with rectal cancer, shorter scan durations can mitigate discomfort and minimize compliance issues associated with prolonged immobility, thereby increasing the likelihood of successful examination. At the same time, a more efficient acquisition workflow enhances MRI system utilization, contributing to improved allocation of healthcare resources and higher patient throughput. Compared with conventional approaches, such efficiency gains may translate into tangible clinical and economic benefits, including a lower risk of repeated examinations, reduced scheduling delays, and expedited patient assessment and treatment decision-making.

Quantitative ADC measurement also plays a pivotal role in the clinical application of DWI. Consistent with previous studies [21], our findings showed that tumor ADC values obtained from rFOV_DL_ DWI were lower than those from fFOV_STA_ DWI. A plausible explanation was that the improved edge sharpness of rFOV_DL_ DWI enabled more accurate delineation of tumor boundaries, thereby minimizing the inclusion of adjacent non-tumorous tissue and reducing partial volume effects. The enhanced sharpness of tumor margins also contributed to improved reproducibility in lesion delineation, resulting in higher inter-reader agreement for ADC measurements. Tumor ADC values derived from rFOV_DL_ DWI showed solid inverse correlations with histopathologic T-staging, underscoring its robustness in assessing tumor aggressiveness. However, tumor ADC values did not show a significant association with N-stage in this study. It is worth noting that, although ADC measurements based on rFOV_DL_ DWI showed promising results in predicting tumor invasiveness, these findings were obtained using a single vendor’s system. The reproducibility of ADC values across different vendors, and even within the same device, remains to be established, which is important for broader application in real-world clinical settings. Therefore, we suggest interpreting our findings with caution when generalizing them to wider clinical contexts.

Our study had several limitations. First, although the overall sample size was reasonable, the number of patients undergoing initial surgery remained relatively small, which might limit the statistical efficacy of the diagnostic analyses. Second, all imaging sequences were acquired in a single center using a single 3.0-T scanner, which may affect the generalizability of our findings. Future multicenter studies involving diverse scanners and vendors are warranted for validation. Third, patients undergoing nCRT or non-curative approaches were excluded, which may restrict the broader applicability of our diagnostic findings. For non-curative cases, pathological staging could not be reliably obtained. Neoadjuvant treatment can introduce complex imaging changes, resulting in restaging that differs from the initial evaluation. Excluding these patients helped maintain a more controlled study cohort. Restaging analyses following nCRT are warranted in future studies. Fourth, the two DWI sequences showed marked visual differences, such as artifact reduction and resolution, which could introduce subjective bias. A crossover reading design would better mitigate this effect. Lastly, we effectively mitigated the high initial noise of rFOV DWI by applying a high-level denoising approach. Still, stronger denoising levels may introduce minor non-linearities in the signal [22], which could potentially affect quantitative analyses derived from these images. The impact of varying denoising levels on *in vivo* quantitative accuracy remains to be explored in subsequent studies.

In conclusion, rFOV_DL_ DWI proved to be highly feasible in the clinical application of rectal cancer. The integration of DLR into rFOV DWI significantly improved image quality while reducing scanning time by 30%. rFOV_DL_ DWI significantly enhanced the primary T-staging accuracy for early-stage tumors (T1–T2) and inter-reader agreements for ADC measurements compared to conventional DWI.

## Supplementary information

**Additional Supplementary Supplement S1:** Extraction of objective image quality metrics and ADC values **Supplement Table S1.** ADC value and its correlation with histopathologic staging.

## Data Availability

The datasets used and analyzed during the current study are available from the corresponding author upon reasonable request.

## Abbreviations

ADC: Apparent diffusion coefficient
DLR: Deep learning reconstruction
DWI: Diffusion-weighted imaging
fFOV: Full field-of-view
fFOV_STA_: Standard-reconstructed full field-of-view
FOV: Field-of-view
ICC: Intraclass correlation coefficient
nCRT: Neoadjuvant chemoradiotherapy
rFOV: Reduced field-of-view
rFOV_DL_: Deep learning-reconstructed reduced field-of-view
SNR: Signal-to-noise ratio
