# Supplementary material for: Deep learning reconstruction accelerated reduced field-of-view DWI in rectal cancer: mucosa-submucosa-muscularis visualization and T staging
Source: Eur Radiol Exp. 2026 Jan 26;10:8. doi: 10.1186/s41747-025-00667-x (PMC12834858; doi:10.1186/s41747-025-00667-x)
Supplement: Supplementary file 1 — Additional Supplementary Supplement S1: Extraction of objective image quality metrics and ADC values Supplement Table S1. ADC value and its correlation with histopathologic staging. [file 41747_2025_667_MOESM1_ESM.pdf]

Deep learning reconstruction accelerated reduced field-of-view DWI in rectal cancer: mucosa-submucosa-muscularis visualization and T staging

ELECTRONIC SUPPLEMENTARY MATERIAL

Supplement S1: Extraction of objective image quality metrics and ADC values

Objective image quality metrics, including signal-to-noise ratio (SNR) of the tumor (SNR_{tumor}), SNR of the internal obturator muscle ($SNR_{internal\ obturator\ muscle}$), and contrast-to-noise ratio (CNR) between the tumor and internal obturator muscle, as well as apparent diffusion coefficient (ADC) values were measured using region of interest (ROI) analysis via the built-in tools on the workstation. Specifically, the reader first identified the largest tumor cross-section on DWI and placed the largest possible circular ROI (area ≥10 mm²) within the tumor. A similarly sized circular ROI (area ≥10 mm²) was placed on the internal obturator muscle in the same slice. The mean signal intensity (SI) within each ROI was recorded as the SI for the respective tissue. The standard deviation (SD) of the SI in the internal obturator muscle ROI was regarded as image noise. Three objective image quality metrics were calculated as follows: (1) $SNR_{tumor} = SI_{tumor} / SD_{obturator\ internus\ muscle}$, (2) $SNR_{obturator\ internus\ muscle} = SI_{obturator\ internus\ muscle} / SD_{obturator\ internus\ muscle}$, (3) $CNR = |SNR_{tumor} – SNR_{obturator\ internus\ muscle}|$. Where SI_{tumor} denotes the mean signal intensity of the tumor, $SI_{obturator\ internus\ muscle}$ denotes the mean signal intensity of the internal obturator muscle, and $SD_{obturator\ internus\ muscle}$ indicates the standard deviation of the signal intensity in the internal obturator muscle. ADC values were measured using the same method from the tumor ROI.

Supplement Table S1. ADC value and its correlation with histopathologic staging

	ADC value (*10^{-3} mm^2/s)	Correlation with T-staging		Correlation with N-staging	
		r-value	*p*-value	*r*-value	*p*-value
**Junior reader**					
$fFOV_{STA}$ DWI	0.962 ± 0.160	-0.446	< 0.001	-0.009	0.934
$rFOV_{DL}$ DWI	0.908 ± 0.143	-0.433	< 0.001	-0.129	0.216
**Senior reader**					
$fFOV_{STA}$ DWI	0.967 ± 0.151	-0.330	0.001	-0.047	0.651
$rFOV_{DL}$ DWI	0.912 ± 0.144	-0.379	< 0.001	-0.114	0.258

The correlation results were analyzed based on the surgery participants (n = 94). ADC values are mean ± standard deviation. *ADC* Apparent diffusion coefficient, *DWI* Diffusion-weighted imaging, $fFOV_{STA}$ Standard-reconstructed full field-of-view, $rFOV_{DL}$ Deep learning-reconstructed reduced field-of-view.
